# Terahertz emission from a spintronic stack nanodecorated with plasmonic nanoparticles

**DOI:** 10.1038/s41598-026-42758-8

**Published:** 2026-03-12

**Authors:** Vittorio Cecconi, Akash Dominic Thomas, Ji Tong Wang, Cheng-Han Lin, Anoop Dhoot, Antonio Cutrona, Abhishek Paul, Luke Peters, Luana Olivieri, Elchin Isgandarov, Juan Sebastian Totero Gongora, Alessia Pasquazi, Marco Peccianti

**Affiliations:** 1 Emergent Photonics Research Centre, Department of Physics, School of Science, Loughborough University, Loughborough , LE11 3TU UK; 2 Department of Information Engineering, Electronics and Telecommunications, Sapienza University of Rome, Rome, 00184 Italy

**Keywords:** Materials science, Nanoscience and technology, Optics and photonics, Physics

## Abstract

Spintronic emitters promise to revolutionise terahertz (THz) sources by converting ultrafast optical pulses into broadband THz radiation without phase-matching constraints. Because the conversion relies on spin-current injection across a nanometre-thin magnetic layer, its efficiency is ordinarily limited by weak optical coupling. Here, we present a demonstration of a drop-casting based approach to introduce ultrafast plasmonic-mediated coupling: a sparse-layer of silica–gold core–shell nanoparticles is deposited directly onto a W/Fe/Pt spintronic trilayer. This sparse (≈ 6%) decoration leads to a measured enhancement of the emitted THz peak field between 1.1x and 1.6x relative to the bare stack as the angle is increased from 0° to 75°, pointing to a very high local conversion enhancement for this low-coverage spintronic emitter compared with the bare stack, with the maximum emission reached at 0°. This demonstration points to a viable pathway toward highly efficient spintronic terahertz emitters with potential applications in spectroscopy, imaging, and ultrafast technologies.

**Supplementary Information:**

The online version contains supplementary material available at 10.1038/s41598-026-42758-8.

## Introduction

Terahertz (THz) radiation (spanning approximately 0.1 THz and 10 THz)^1^ has become increasingly pervasive across scientific and industrial domains, with applications in communications^2,3^, sensing and non-destructive testing across several sectors.^4^.

Progress in these domains is ultimately tied to the development of efficient and scalable THz sources. State-of-the-art approaches, based on various optical-to-THz conversion mechanisms, generally present distinct trade-offs in efficiency, bandwidth, and platform compatibility^1,4^.

Photoconductive emitters, characterised by high brightness^5^ and modest optical excitation requirements, currently dominate THz spectroscopy applications but do not offer a route for surface scalability or broadband emission. Benchmark nonlinear emitters are based on transparent optical crystals such as ZnTe, LiNbO₃, and DAST and generate THz pulses primarily via optical rectification^6^. Owing to their high saturation threshold, they can achieve very high pulse energies and represent a standard technological deployment. However, these remain fundamentally bulk systems, with limits in integration, scalability and bandwidth, as spectral performance is constrained by phase-matching requirements and by phonon-driven absorption (e.g., Reststrahlen band). As an alternative option, semiconductor surface emitters, e.g., low-bandgap media like InAs and InSb, rely on carrier-driven emission mechanisms, enabling high conversion efficiency per unit thickness^7^. Compared to transparent emitters, they benefit from established fabrication processes in the electronics domain, allowing low-cost area scalability. However, they offer low pump saturation^8^ and low THz transparency with absorption dominated by Drude free-carrier loss.

Spintronic terahertz emitters (STEs) represent a recent pivotal evolution in overcoming the scalability limits of bulk THz generation technologies. Based on simple nanoscale multilayer structures, they offer phase-matching-free broadband spectral emission with essentially no gaps^9–12^. Over the past decade, their adoption has grown rapidly, accompanied by intense efforts to understand and control the underlying spintronic physics^13–16^. THz radiation from spintronic emitters originates from the excitation of spin-polarised hot electrons in a ferromagnetic (FM) layer by an ultrafast laser pulse. In the most common operating mechanism, the optical excitation promotes majority-spin carriers into higher-energy, more delocalised orbitals, generating a diffusive spin current^17–19^. This current is then injected into a non-magnetic (NM) metallic layer, where it is converted into a transverse charge current via a spin–charge conversion mechanism^20,21^, such as the inverse spin Hall effect (ISHE)^22^.

In a typical high-efficiency embodiment, the emitter consists of a sandwich structure where the FM layer is embedded between two NM layers with large (and opposite) spin Hall angles (e.g., tungsten and platinum). The structure operates under an external static magnetic field that orients and saturates the FM magnetisation^9,22^. A significant research effort has focused on the dependence of THz emission on the FM and NM layer thicknesses and interfacial properties as optimisation parameters^20^ and has shown that STEs can reach emission efficiencies comparable to benchmark nonlinear crystals (e.g., ZnTe)^9^. Despite these advances, optical–FM coupling remains fundamentally limited by the trade-off imposed by the limited diffusion lengths of spin currents, with optimisation often requiring demanding sub-nm control of layer growth^23,24^. Such ultrathin active regions inherently limit the energy transfer from the excitation^25^.

To address the limited optical-to-THz conversion efficiency, plasmonic-mediated light coupling has been proposed as a route to enhance the chain of physical mechanisms involved in THz generation. Explored solutions include the use of Au nanoparticles fabricated via high-temperature annealing of an ultrathin Au layer. These particles are subsequently embedded within the ferromagnetic (FM) layer^26^, enabling the surface plasmons to act as a spin-pump^27^. On the other hand, the idea of introducing field-localisation via engineered optical resonators by surface sparse deposition has been seminally explored by Liu and co-workers^28^, who demonstrated THz emission enhancement through the deposition of a bulk layer of gold anisotropic nanorods atop a spintronic trilayer. These approaches tend to be scalable and focus on engineering plasmonic nanoresonators to maximise the local field and its coupling from the surface of the spintronic stack.

More broadly, a large variety of surface nanostructures have been explored for nonlinear responses. These include absorptive metasurfaces^29–31^, as well as metallic and plasmonic resonators^32–37^, and surface-deposited distribution of resonant nanoparticles, which allows effective tailoring of optical resonances and enhanced local field intensities^38^. Within this context, sparse or self-assembled nanostructures offer an appealing route that combines field enhancement with scalable and straightforward fabrication strategies^32,38–41^. For example, silicon surfaces textured into random “black silicon” needle arrays have shown significantly stronger THz emission under ultrafast optical excitation compared to unstructured substrates^42^.

**Fig. 1:**
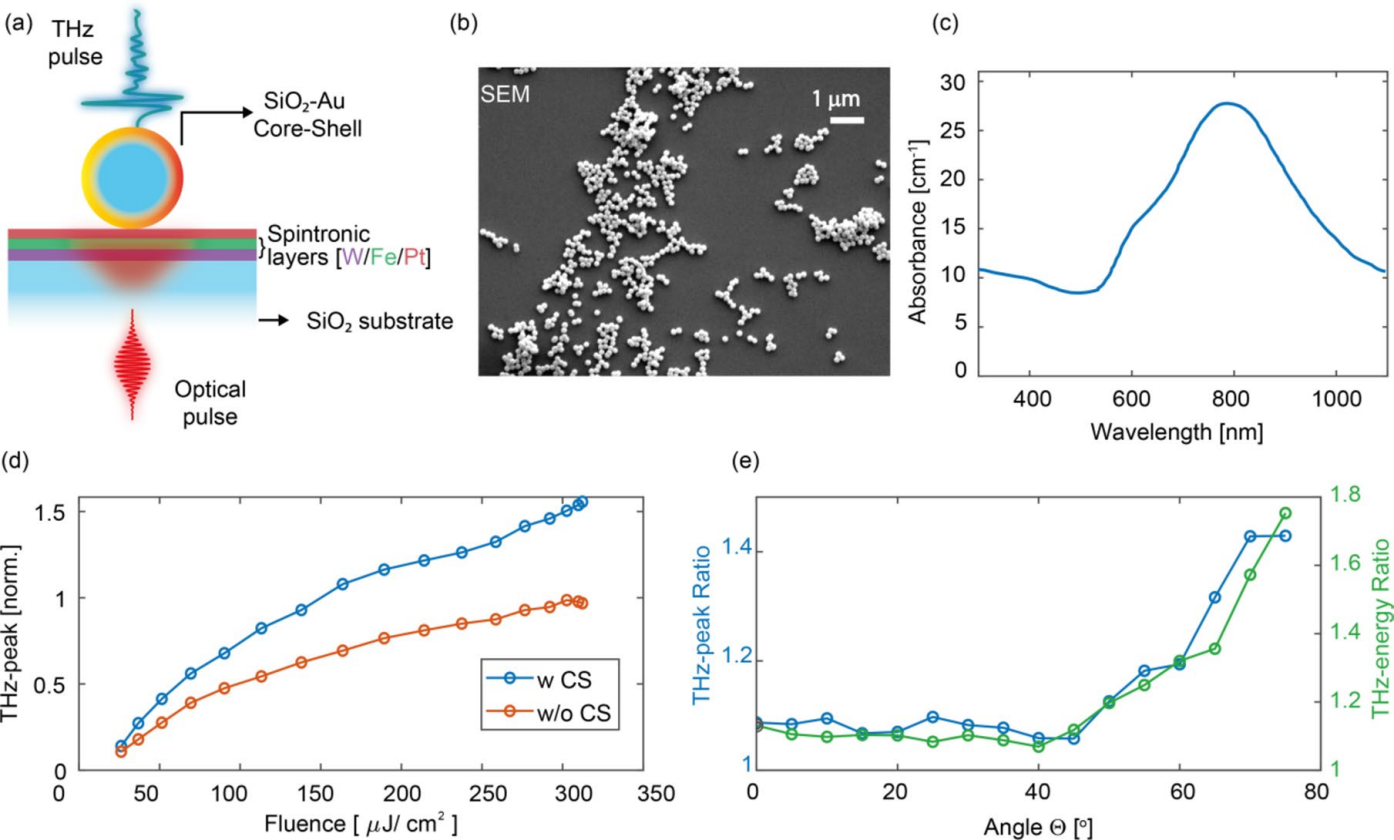
Illustration of the structure and operating principle of the plasmonically-enhanced spintronic assembly. (**a**) Schematic of the plasmonically-enhanced THz spintronic emitter (STE), consisting of a core–shell nanoparticle positioned on a spintronic trilayer structure composed of W/Fe/Pt, each layer 2 nm thick. (**b**) SEM image of the silica-gold core-shell nanoparticles deposited onto the spintronic stack. (**c**) Optical properties of the core-shell nanoparticle solution with a peak light absorption around 800 nm (data from nanoComposix Inc. for particles delivered in a water solution); the gold-shell silica-core structure has a diameter of 154±7 nm. (**d**) Experimental THz emission enhancement as a function of pump power for samples with (blue) and without (red) CS nanoparticles at an incidence angle of Θ=75° - data are normalised relative to the reference THz peak-value at the pump peak fluence ~316 µJ/cm^2^ (390 mW). (e) THz enhancement (peak field and total pulse energy, with and without CS) vs. angle of incidence at a fixed power of 390 mW.

In this work (Fig. 1a), we introduce an ultrafast plasmonic-heating strategy to push spintronic terahertz emitters decisively beyond the established optical–FM coupling limit. By drop-casting a sparse monolayer of Au–SiO₂ core–shell nanoparticles (CS) directly onto a nanometre-thick W/Fe/Pt trilayer deposited onto a silica substrate, we exploit localised surface-plasmon resonances^43^ to funnel pump energy into nanometric hot spots projected onto a spin-polarised magnetic core.

Figure 1b shows a typical SEM (a full SEM diagnostics is proposed in the supplementary material) image we used to estimate the area coverage of the particles exhibiting monolayer clusterisation and very modest percolation. The average coverage is fully determined by the density of the initial solution.

In our experiment, an average field enhancement ranging from 1.1x to 1.6× is observed as the impinging angle is increased towards Θ = 75° on the glass substrate (Fig. 1d), for a fixed pump fluence. While clusterisation makes the local enhancement heterogeneous, the locality of the excitation suggests a strong overall local field-emission enhancement. Under a fully-local, coherent superposition assumption, from a simple estimate we can infer a field enhancement of approximately 2.7x at 0° and 11x for the covered areas at large angles compared to the case with no CS.

Interestingly, this is done without exerting any complex control of the deposition, beyond the solution density. We highlight that the comparison of the enhancement vs. the angle in Fig. 1 is proposed at constant fluence and not at constant power, to keep the local excitation on the spintronic substrate surface similar. This means that the pump power increases as the illuminated area (beam footprint) increases with the incidence angle. This result also offers context to previous explorations (e.g in ref.^28^ 77% enhancement is observed with anisotropic plasmonic nanorods).

**Fig. 2:**
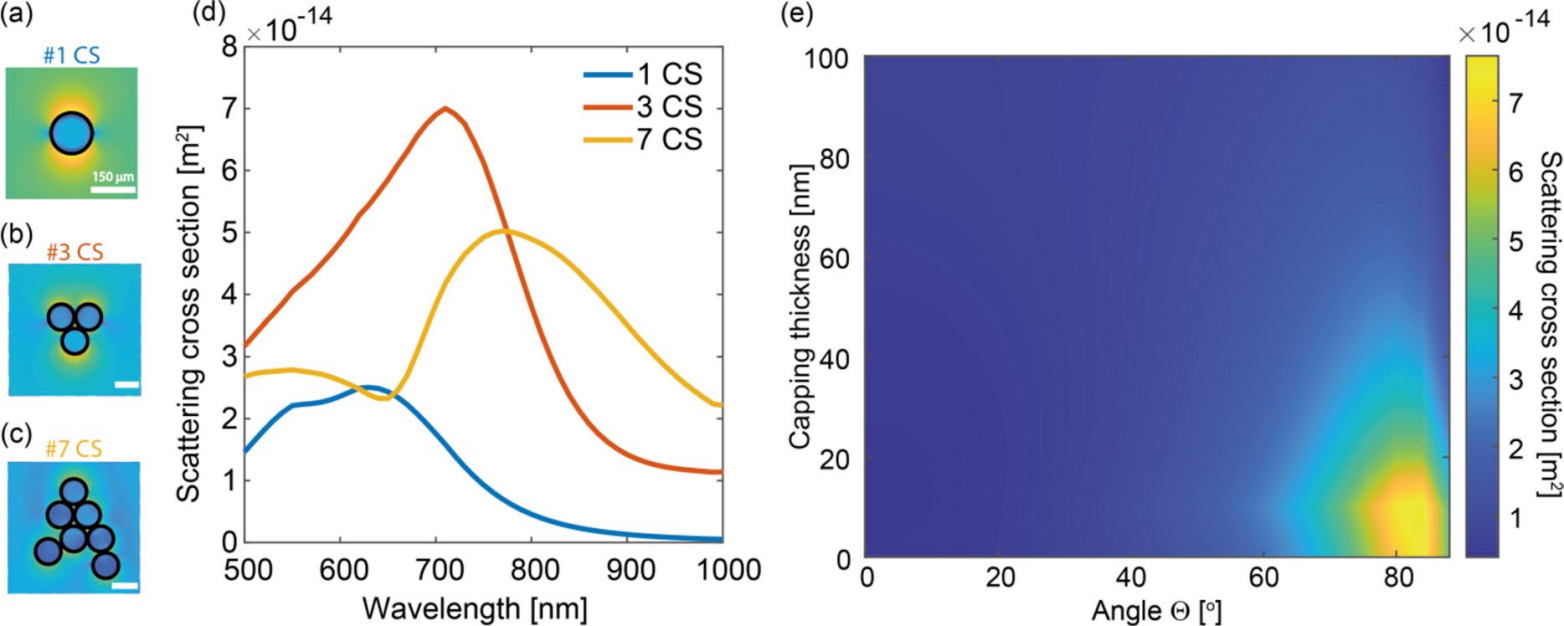
Simulated scattering cross section results and capping thickness study, for a sample illuminated through the glass substrate (**a-c**). The field value at the middle plane of the core-shell. The black lines highlight the boundaries of the nanoparticles. (**d**) Nanoparticle-induced scattering cross-section of a nanometric platinum layer for three configurations: a single particle, a three-particle system, and a cluster of seven nanoparticles. The results show that for the seven-particle case, the spectrum of the trilayer spintronic broadens, indicating an overall enhancement in tunability. (**e**) Scattering cross-section efficiency as a function of silica capping thickness and angle of incidence for a p-polarised wave. The scattering cross-section is defined as the ratio of the outwards radiated scattered power and the impinging intensity.

In our case, we deploy rotationally invariant plasmonic resonators, i.e., the local alignment of the particle is not required for the overall effect, and for this geometry, the enhancement remains robust even with significant clusterisation (Fig. 2). This is quite relevant in comparison with dipole-like resonators. The mutual alignment of nanorods or other anisotropic structures, and their relative alignment to the light polarisation, are both relevant in pursuing field enhancement. Invariance to rotation is indeed an important aspect when coarse deposition approaches are sought.

At higher densities, additional effects such as near-field coupling and optical shadowing are expected to modify the scaling. When strong nanoresonator coupling occurs, the local enhancement might decrease.

## Results

The CS nanoparticles act as nanoscale resonators^44^, enhancing the local optical intensity, projecting a mode-hot spots that overlap with the W/Fe/Pt spintronic trilayer (Figs. 1a, b). Figure 1b presents a scanning electron microscope (SEM) image of the spintronic surface after deposition, clearly revealing a sparse distribution. The optical response of the nanostructure is resonant at our Ti: Sa pump central wavelength (800 nm). Each core–shell particle has a total diameter of 150 nm, consisting of a silica core surrounded by a 20 nm-thick gold shell. A typical advantage of the CS geometry is the ability to exhibit peaked resonant enhancement in the near-IR, compared to solid nanoparticles^45^. The larger size is beneficial for direct deposition, as it reduces percolation (being also safer to handle and process). The resonance bandwidth accessible with this design is typically compatible with 100 fs-scale ultrafast laser excitations.

In the exploited structure, the core mechanism of spintronic THz emission involves the inverse spin Hall effect (ISHE)^46^, which converts optically-injected spin current into a charge current, with a local current density ${J}_{c}$ generally governed by

1 $${J}_{c}={\theta}_{SH}\left({J}_{s}\times\sigma\right)$$

where ${\theta}_{SH}$ is the spin Hall angle (a measure of spin-orbit coupling strength), ${J}_{s}$ is the spin current density, and $\sigma$ is the spin polarisation direction. The charge current is orthogonal to both the spin current and the spin polarisation vector, determined by the magnetic polarisation of the FM medium. The generated charge current acts as a time-varying dipole, which radiates electromagnetic waves in the THz frequency range. Model-wise, the emitted THz electric field ${E}_{THz}$ is proportional to the time derivative of the charge current density

2 $${E}_{THz}\left(t\right)\propto\frac{\partial{J}_{c}\left(t\right)}{\partial t}$$

The optical coupling of the CS-layer with the spintronic trilayer is strongly influenced by the spatial configuration of the core–shell nanoparticles. We numerically simulated how particle clustering induces a spectral shift and broadening in the scattering cross-section for a sample illuminated through the glass substrate. For the core-nanoshell, our numerical estimation shows enhancement that remains effective across various configurations (Fig. 2). The corresponding electric field distributions at 800 nm for varying morphology (Fig. 2a-c) reveal that in general the local field profile is shaped by both the interaction with the impinging light (normal in Fig. 2a-c) and near-field coupling between adjacent nanoparticles, although localisation always occurs. Considering moderate clustering (e.g., seven particles, consistent with the general observation from SEM images across our samples), the scattering cross-section around the pumping wavelength is effective in several configurations (Fig. 2d).

We also assessed the role of the distance between the core–shell nanoparticles and the spintronic trilayer in achieving optimal coupling, specifically by introducing a dielectric capping layer as a spacer. Figure 2e shows how varying the thickness of a silica capping layer affects the scattering cross section of the CS nanoparticles. The analysis reveals that increasing the capping thickness leads to a pronounced reduction in the scattering cross section, particularly for p-polarised waves at high incidence angles (Fig. 2e), supporting our design choice to omit any capping to maximise coupling. Notably, the application of nanometric capping layers is widespread in ultrathin device engineering, typically for environmental protection^47,48^. However, the topmost layer in our spintronic stack is platinum, which is known for its high chemical stability and relative resistance to environmental degradation, particularly compared to more reactive metals.

**Fig. 3:**
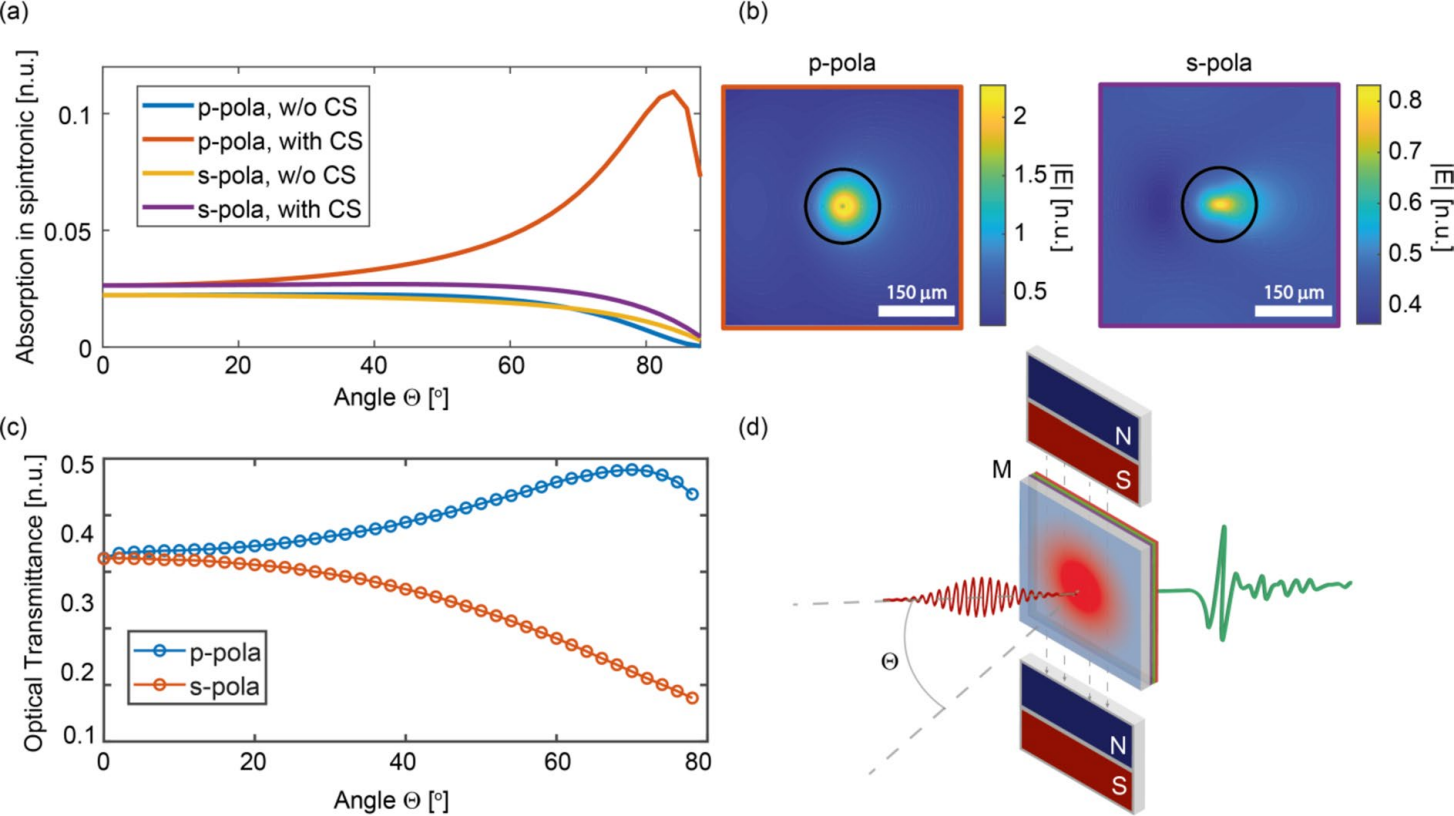
Numerical and experimental optical response of CS nanoparticles (**a**) Simulated absorption of the optical pump in the trilayer spintronic structure as a function of incidence angle. (**b**) Simulated electric field distributions at the spintronic plane for p- and s-polarised light at an incidence angle of 84°. The black circle denotes the area over which the absorption is calculated in (a). (**c**) Experimental optical transmission spectra of the spintronic sample (without CS) at various angles of incidence on the glass substrate, comparing s- and p-polarisations. (**d**) Schematic of the experimental setup. The sample is immersed in a magnetic field M and rotated in the orthogonal plane, with an angle Θ between the beam and the sample surface.

In Fig. 3a, our numerical calculation predicts a pronounced angular dependence in the absorption of the optical pump within the spintronic trilayer when placed in direct contact with the CS layer. For p-polarised light, absorption is markedly enhanced at large incidence angles, which in turn leads to increased THz emission. Simulated electric field distributions at the spintronic interface further confirm the superior field confinement achieved for p-polarised light at high incidence angles, in contrast to the s-polarised case (Fig. 3b). The red plot in Fig. 3a suggests that, under our experimental conditions, for p-polarised light, the THz enhancement should increase with the angle of incidence.

Notably, Fig. 3c shows the experimental measurements of the optical transmission of the spintronic stack across both polarisations. In particular, the data show that the overall transmission of the p-polarisation increases at high angles, both due to the overall reduction of the absorption and impedance matching, corresponding to a general reduction of the local conversion efficiency.

The measurement setup (Fig. 3d) enables a systematic investigation of the angular dependence of THz emission via rotation of the sample within a controlled static magnetic field (~ 100 mT), with the field direction orthogonal to the rotation axis (see Supplementary Information for schematic details). In this configuration, the sample is illuminated by a Ti: Sa regenerative amplifier (800 nm, 1 kHz, 76 fs pulses, 9 mm 1/e² beam diameter), while the incidence angle Θ between the laser and the sample surface is varied over a wide range (see Supplementary Information for further details). The THz field amplitude is then recorded as a function of Θ (Fig. 1e).

**Fig. 4:**
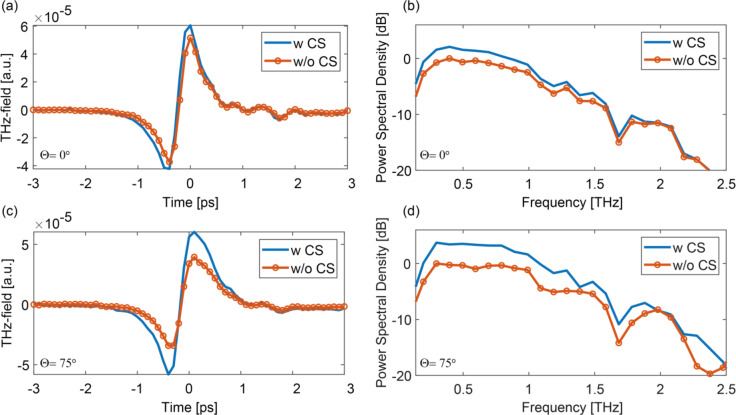
Experimental THz enhancement angle-dependence campaign (**a**,** b**) Time-domain trace and corresponding frequency spectrum of the THz field at normal incidence ($\Theta$ = 0°); (**c**,** d**) Time-domain trace and corresponding spectrum $\Theta$ = 75°. All measurements were performed at a constant pump peak fluence of around 300 µJ/cm^2^ (which also means that the overall power increases with the angle of incidence).

Representative detected THz time-domain waveforms and their corresponding frequency spectra at Θ = 0° and Θ = 75° are shown in Fig. 4, highlighting the emission enhancement induced by the CS-layer (with the latter showing a greater enhancement).

## Discussion

**Fig. 5:**
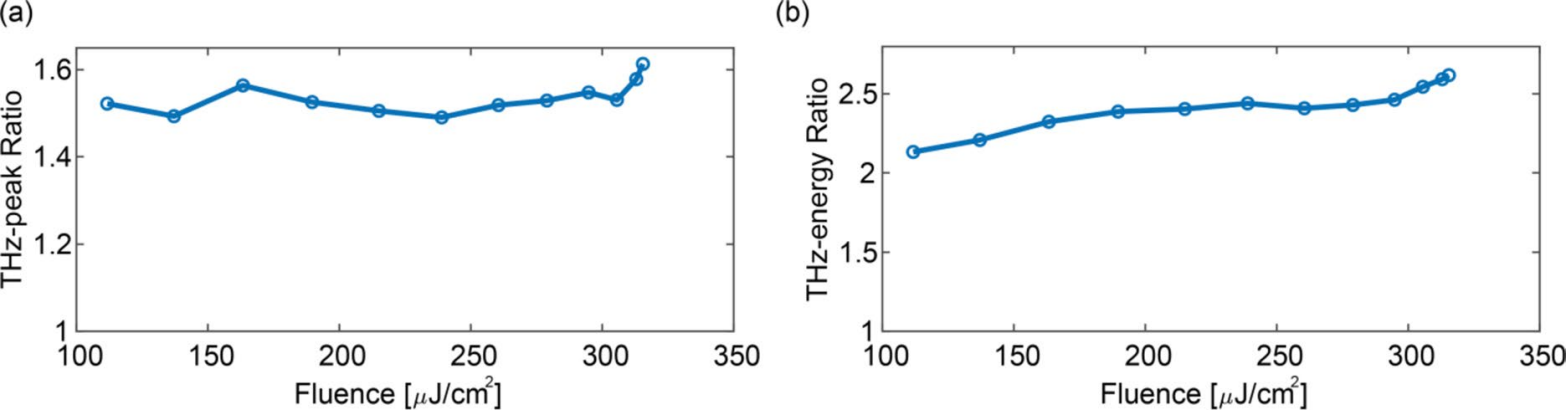
THz enhancement trends: (**a**) Enhancement in THz peak field amplitude due to the CS layer, plotted as the ratio of the peak fields with and without CS, as a function of pump peak fluence. (**b**) Enhancement in THz pulse energy, shown as the ratio of pulse energies with and without CS, versus pump peak fluence. All measurements were taken at $\Theta$ = 75°.

Figure 5 illustrates the dependence on the fluence of the enhancement induced by the CS layer. The ratio of THz peak field amplitudes with and without the CS layer increases with pump fluence, reaching up to 1.6 in the explored range (Fig. 5a). The corresponding trend for THz pulse energies is shown in Fig. 5b, showing a growing enhancement across the range. The plasmonic CS layer provides a substantial boost to the overall conversion efficiency. Notably, the fluctuations observed are primarily attributed to airflows in our experimental setup, as temperature variations in the spintronic stack substrate can perturbatively affect the conversion efficiency (see Supplementary Information).

Interestingly, at the explored fluence levels, saturation effects become significant, leading to deviation of the response from a purely quadratic dependence as a result of both local saturation and thermal derating (i.e., reduced efficiency at elevated temperatures).

Nevertheless, the observed enhancement persists as the excitation fluence increases and is more pronounced at Θ = 75°.

**Fig. 6:**
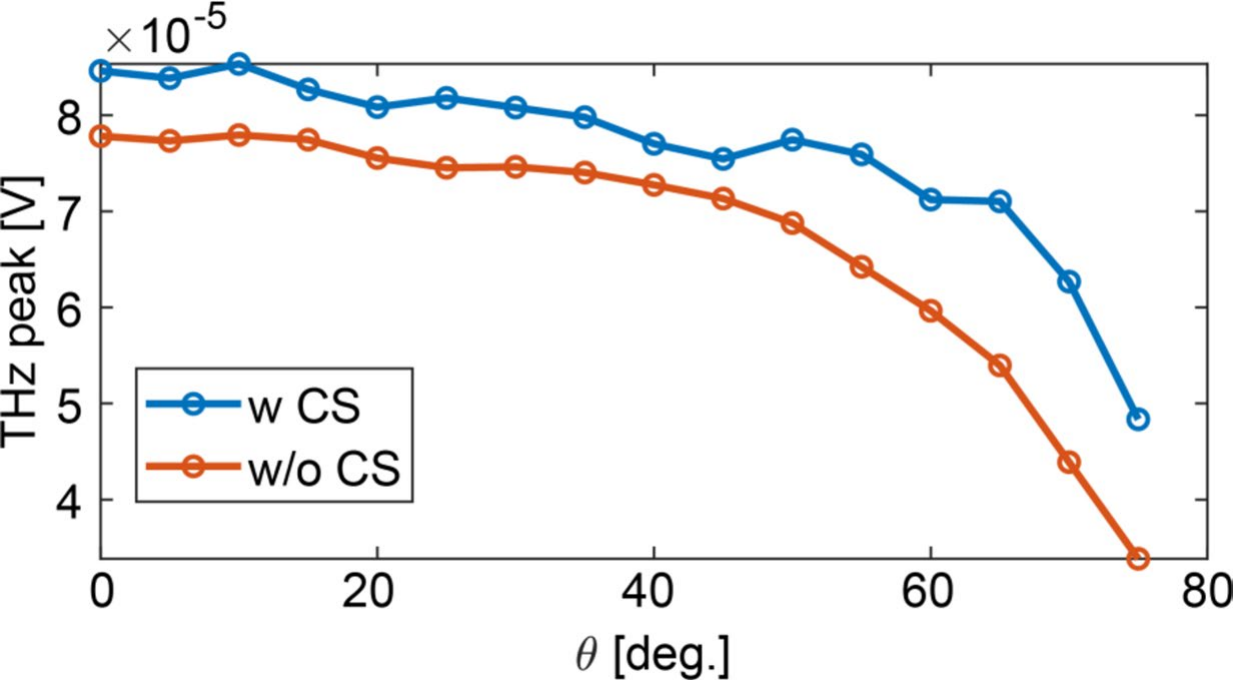
Dependence of the THz peak field on the angle between the pump beam and the surface normal of the spintronic sample at constant power. The ratio between the peak field of the sample emission with and without the core–shell nanoparticles grow with the angle.

Figure 6 shows that the absolute THz peak field (measured at constant pump power) is maximised at normal incidence. This follows the intrinsic emission trend versus angle of our reference STE stack used and is apparently not correlated to the CS nanodecoration. Consistent with Fig. 1e, we can appreciate that the enhancement is progressively larger with the angle from 1.1x to 1.6x in our scan. In a reference quantitative comparison within sparse plasmonic-assisted STEs, Liu et al.^28^ reported up to 77% enhancement at normal incidence using a layer of Au nanorods, although the role of the coverage is not directly addressed in that work.

## Conclusion

Given the considerable attention devoted in the literature to the optimisation of spintronic stacks, we believe nanodecoration with CS is potentially enabling in the exploitation of STEs, with a very limited technological overhead. Although extensive quantitative benchmarks are beyond the purpose of this work, these observations suggest that the optimisations toward structures with surface nano-resonator-mediated coupling are potentially more rewarding than simply maximising the emission from the bare stack. Interestingly, our SEM images (see supplementary information) suggest that this coverage and nano-shell clustering (typical of dense layers) are not a significant hindering element in the enhancement. In essence, CS-enhanced spintronic emitters provide access to local efficiencies largely exceeding that of the native structure.

In conclusion, we show that the combination of plasmonic core-shells with a thin spintronic platform affects the overall conversion efficiency of STEs. By depositing a sparse monolayer of Au–SiO₂ CS nano-resonators on the spintronic layer, the nanoparticles act as ultrafast plasmonic couplers leading to a dramatic increase in the near-field coupling within the FM-layer, promoting terahertz generation. The macroscopic THz field enhancement achieved with very low surface coverage suggests very high local conversion enhancement. We believe this represents a key step toward new optimisation pathways in STE technology.

## Materials and methods

The sample layout consists of a core–shell nanoparticle positioned on a spintronic trilayer composed of tungsten, iron, and platinum, each with a thickness of 2 nm, deposited on a 1 mm thick SiO₂ substrate - BOROFLOAT^®^ 33 (NEXTERION^®^ Glass B) - and deposited using DC-magnetron sputtering or e-beam sputtering under a working gas pressure below 7*10^− 7^ Torr. Details for the fabrication process are provided in the Supplementary Information.

The nanoparticles are randomly distributed across the surface. The SEM characterisation (Fig. 1b and Fig. S4) shows a general monolayer-clustered distribution of CS with an average relative surface coverage of approximately 6% (see table in Supplementary Information for a more detailed analysis of this estimation). The CSs (from nanoComposix Inc.) have a gold-shell silica-core structure with a diameter of 154 ± 7 nm (a silica core of diameter 117 ± 7 nm with a gold shell of thickness 19 nm) and feature a peak absorption centred at around λ = 800 nm (Fig. 1b), compatible with our Ti: Sa laser pump source (data from nanoComposix Inc.). The deposition step is performed via drop-casting of a dispersion in a solution of concentration 0.05 mg/mL.

## Supplementary Information

Below is the link to the electronic supplementary material.

Supplementary Material 1

## Data Availability

All data required to reproduce the results published in the main text are available via Figshare ( [https://doi.org/10.6084/m9.figshare.29518838](https:/doi.org/10.6084/m9.figshare.29518838) ).
