# Supplementary material for: Terahertz emission from a spintronic stack nanodecorated with plasmonic nanoparticles
Source: Sci Rep. 2026 Mar 12;16:13311. doi: 10.1038/s41598-026-42758-8 (PMC13106760; doi:10.1038/s41598-026-42758-8)
Supplement: Supplementary file 1 — Supplementary Material 1 [file 41598_2026_42758_MOESM1_ESM.pdf]

Supplementary Material: Terahertz Emission from a Spintronic Stack Nanodecorated with Plasmonic Nanoparticles

Vittorio Cecconi,[1,2] Akash Dominic Thomas,[1] Ji Tong Wang,[1] Cheng-Han Lin,[1] Anoop Dhoot,[1] Antonio Cutrona,[1] Abhishek Paul,[1] Luke Peters,[1] Luana Olivieri,[1] Elchin Isgandarov,[1] Juan Sebastian Totero Gongora,[1] Alessia Pasquazi,[1] and Marco Peccianti[1]

[1]*Emergent Photonics Research Centre, Department of Physics, School of Science, Loughborough University, LE11 3TU, UK*

[2]*Department of Information Engineering, Electronics and Telecommunications, Sapienza University of Rome, 00184 Rome, Italy*

SPINTRONIC CHARACTERISATION

Spintronic Benchmark against $< 110 >$-ZnTe emitter

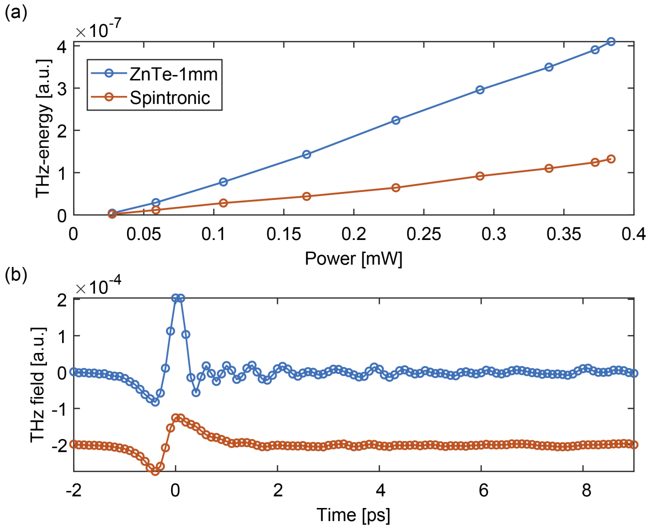

FIG. S1. Benchmark of THz emission from the native spintronic sample against a <110>-oriented 1-mm-thick ZnTe crystal. (a) Emitted THz energy as a function of pump power for both the spintronic emitter and ZnTe reference. The spintronic emission reaches approximately one-fourth the energy of the ZnTe crystal, comparable to a ∼0.5-mm-thick ZnTe emitter. (b)Time-domain THz waveforms acquired from each emitter at pumping power $P = 166$ mW .

To quantitatively benchmark the performance of our spintronic terahertz (THz) emitter, we directly compared its emission characteristics to those of a standard <110>-oriented 1-mm-thick ZnTe crystal under identical excitation conditions. As shown in Fig. S1a, the THz energy emitted by the spintronic sample is approximately one-fourth of that measured from the ZnTe crystal across the explored range of pump powers. This suggests that the emission efficiency of our spintronic sample is comparable to that of a ∼0.5-mm-thick ZnTe crystal, with the efficiency of the core-shell enhanced sample quite exceeding this level. Figure S1b presents the corresponding time-domain THz waveforms.

Vibrating sample magnetometry measurement

To determine the magnetic field required to fully saturate the spintronic layer, we performed Vibrating Sample Magnetometry (VSM) on the same platform used in our core–shell experiments. The resulting VSM data provide the magnetic response of the sample, measuring the magnetisation M as a function of the applied field H. By sweeping H from positive to negative saturation, we observed a well-defined hysteresis loop (see Fig. S2)

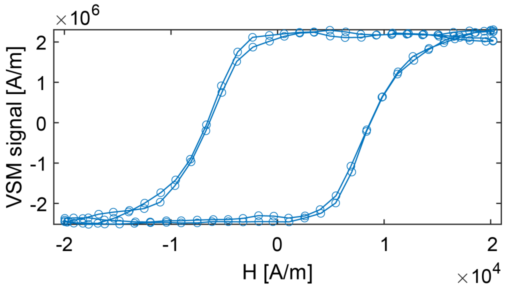

FIG. S2. VSM measurement of the spintronic sample.

with a coercivity of approximately 7.5, kA/m and saturation achieved for fields above approximately 20, kA/m. Based on this result, we used Neodymium N42 permanent magnets in our experimental setup to generate an in-plane magnetic field exceeding 30mT.

X-ray reflectivity measurement

The layered-structure of the W/Fe/Pt spintronic emitter was characterised using a Siemens D5000 x-ray diffractometer with a Cu Kα source (0.15418 nm). X-ray reflectivity ($\theta/2\theta$) measurements were conducted by scanning the spintronic film at incident angles ranging from 0.4° to 10°, with a step size of 0.01°. The operation voltage of 40 kV and current of 30 mA were set at two scanning ranges of 0.4°-1° and 2°-10°, while 30 kV and 10 mA were used for the 1°-2°, respectively. Structural parameters of the W/Fe/Pt tri-layer, including thickness t, RMS roughness σ, and density ρ, were extracted by fitting the reflectivity intensity curve using the GenX

software [1]. The substrate/W/Fe/Pt structure was first modelled with reference density values. The derived parameters for the individual layers in the tri-layer spintronic emitter are shown in Table S1. The corresponding XRR data plot is presented in Fig.S3a and SLD profile in Fig.S3b.

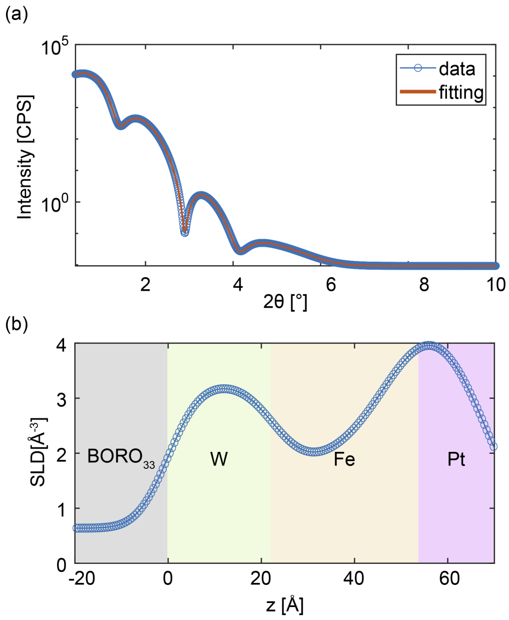

FIG. S3. (a) XRR data (red circles) with the simulated fit (black line) for the W/Fe/Pt spintronic trilayer. (b) Corresponding scattering length density (SLD) profile illustrating the depth-dependent structure of the spintronic trilayer.

TABLE S1. XRR fitting parameters for the W/Fe/Pt spintronic tri-layer were obtained using GenX reflectivity software.

Layer	Thickness t (nm)	Density σ (atoms/Å^{-3})	Roughness ρ (nm)
Pt	1.64	0.0828	1.03
Fe	2.74	0.0735	0.994
W	2.25	0.0473	0.540
BOROFLOAT® 33	1×10^6	0.021	0.533

SPINTRONIC THERMALISATION DYNAMICS

We investigate the thermal response of the spintronic emitter under optical pumping and its influence on THz emission. The results, shown in Fig.S4, present a time-resolved evolution of the THz peak amplitude as the sample transitions from a cold to a thermally equilibrated state. Measurements were conducted at both normal incidence (0°, Fig.S4b) and oblique incidence (75°, Fig.S4a). We observe that the THz emission stabilises after a few minutes, indicating that the sample has reached thermal equilibrium. Due to the variation in pump fluence between the two angles, the difference in THz peak amplitude between the cold and thermalised states is more pronounced at 0° incidence than at 75°, where the effective fluence is lower.

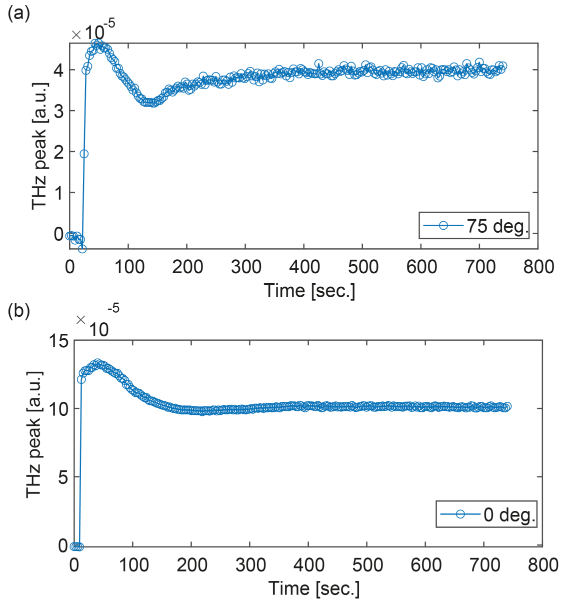

FIG. S4. Spintronic Emission vs illumination time at (a) $\Theta = 0°$ and (b) $\Theta = 75°$ at power 390mW

NANOPARTICLES COVERAGE

We analysed multiple Scanning Electron Microscope (SEM) images to determine the distribution of nanoparticles present in each image and extracted the mean coverage using K-Means clustering. K-Means [2] is an unsupervised clustering algorithm that groups similar data points based on intensity values. It segments the image into two clusters: the background pixels (namely, the lower intensity values), and the nanoparticle pixels (with higher intensity values).

To determine the cluster centres, the algorithm calculates the mean intensity of each cluster. A threshold is then set at the midpoint between these two means to classify pixels as either nanoparticles or the background. The method then counts the white pixels (nanoparticles) and computes the percentage coverage using the formula:

$$\text{Coverage} = \frac{\text{Nanoparticle Pixels}}{\text{Total Pixels}} \times 100$$

This method allows us to efficiently and consistently quantify nanoparticle coverage across a large set of SEM images. By analysing 29 images, we determined that the average surface coverage is 6±3 % (see Fig.S5), indicating a relatively sparse distribution of nanoparticles with spatially distributed plasmonic ‘hot spots’. These findings

suggest that the significant THz field enhancement observed in our experiments arises from exceptionally high local efficiency within the plasmonic regions.

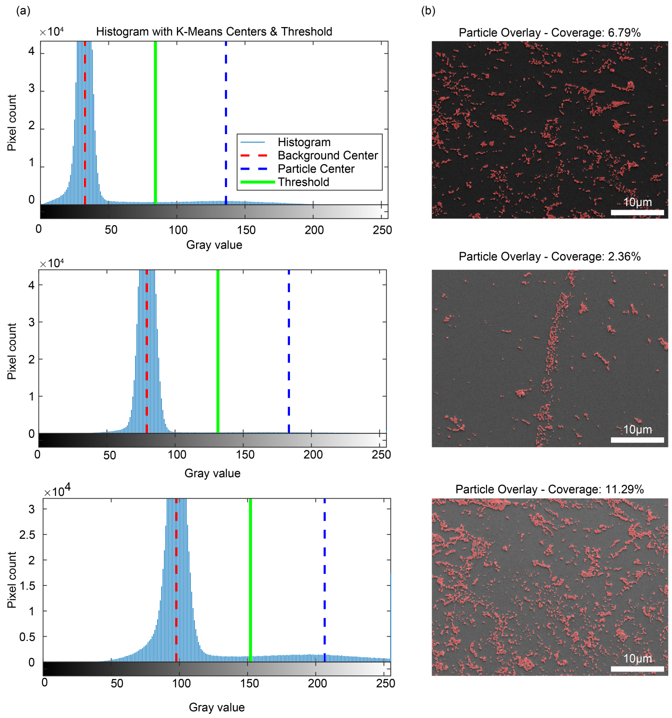

FIG. S5. (a) Histogram of grayscale intensities with K-Means cluster centres (background and particle) and the computed threshold indicated. (b) Overlay of detected nanoparticles (in red) on the original image, highlighting the area coverage percentage and the almost perfect identification of the nanoparticle coverage. SEM images of the nanoparticles deposited on the surface of the spintronic sample reveal a notably sparse distribution, with the majority forming a monolayer.

MULTIPOLE DECOMPOSITION OF PLASMONIC RESPONSE

The numerical simulations of the core–shell nanoparticle presented in Figs. 2-3 of the main text reveal giant near-field resonant effects arising from localised surface plasmon resonances. To gain deeper insight into the optical properties of a single core–shell nanoparticle, we analyse the origin of its resonant features by performing a multipole expansion under Cartesian coordinates [3].

Specifically, we start by expressing the relation between the local electric field and the induced current density within the nanoparticle as $\mathbf{J} = i\omega\epsilon_0\,(\tilde{\epsilon}_r - 1)\,\mathbf{E}$, where ω is the angular frequency, ϵ_0 is the free-space permittivity, and $\tilde{\epsilon}_r$ is the complex relative permittivity describing both the core and gold shell. We can then enumerate the different multipole moment contributions as:

Electric dipole moment:

$$\mathcal{P} = \frac{1}{i\omega}\int d^3r\,\mathbf{J} \tag{S.1}$$

Magnetic dipole moment:

$$\mathcal{M} = \frac{1}{2c}\int d^3r\,(\mathbf{r}\times\mathbf{J}) \tag{S.2}$$

Toroidal dipole moment:

$$\mathcal{T} = \frac{1}{10c}\int d^3r\,\left[(\mathbf{r}\cdot\mathbf{J})\mathbf{r} - 2r^2\mathbf{J}\right] \tag{S.3}$$

Electric quadrupole moment:

$$Q^{(e)}_{\alpha\beta} = \frac{1}{i2\omega}\int d^3r\,\left[r_\alpha J_\beta + r_\beta J_\alpha - \frac{2}{3}(\mathbf{r}\cdot\mathbf{J})\delta_{\alpha\beta}\right] \tag{S.4}$$

Magnetic quadrupole moment:

$$Q^{(m)}_{\alpha\beta} = \frac{1}{3c}\int d^3r\,\left[(\mathbf{r}\times\mathbf{J})_\alpha r_\beta + (\mathbf{r}\times\mathbf{J})_\beta r_\alpha\right] \tag{S.5}$$

where c is the speed of light in vacuum and $\alpha, \beta = x, y, z$.

The total scattered power into the far-field is the sum of the contribution from all multipoles as

$$P = P_{\mathcal{P}} + P_{\mathcal{M}} + P_{\mathcal{T}} + P_{Q^{(e)}} + P_{Q^{(m)}}$$

where,

$$P_{\mathcal{P}} = \frac{\omega^4}{12\pi c^3}|\mathcal{P}|^2 \quad P_{\mathcal{M}} = \frac{\omega^4}{12\pi c^3}|\mathcal{M}|^2 \quad P_{\mathcal{T}} = \frac{\omega^6}{12\pi c^5}|\mathcal{T}|^2$$
$$P_{Q^{(e)}} = \frac{\omega^6}{160\pi c^5}\sum_{\alpha\beta}|Q^{(e)}_{\alpha\beta}|^2 \quad P_{Q^{(m)}} = \frac{\omega^6}{160\pi c^5}\sum_{\alpha\beta}|Q^{(m)}_{\alpha\beta}|^2$$

The core-shell nanoparticle is numerically modelled and simulated using finite-element method as a silica core [4] with radius of 58 nm and gold shell with thickness of 19 nm, consistent with the experimental setting. The spintronic structure is modelled as a multilayer with 2 nm platinum, 2 nm iron, and 2 nm tungsten [5]. Consistently with our analysis in Fig. 2 of the main text and with the experimental procedure, we excite the particle with a linearly polarised, normally incident plane wave. As shown in Fig.S6, the multipole analysis reveals that the electric dipole response dominates the scattering properties of the core-shell nanoparticle over a wide wavelength range, spanning from 500 nm to 1000 nm. The field-superposition of all the responses conflates in the cross-section "1 CS" in Fig. 2d, for an isolated nanoshell. Fig. 2a evidences the typical features of the electric-dipole type resonances, characterised by two clear hot spots along the field polarisation axis around the nanoparticle boundary with dipole direction aligned with the incident field polarisation.

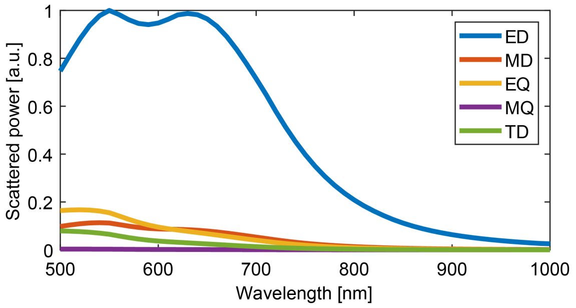

FIG. S6. Numerical characterization of optical properties of core-shell nanoparticle calculated scattered power for the dominant electromagnetic multipoles induced in the nanoparticle. ED: electric dipole; MD: magnetic dipole; TD: toroidal dipole; EQ: electric quadrupole; MQ: magnetic quadrupole.

EXPERIMENTAL SETUP

In Figure S7 we show the experimental setup schematic for a terahertz time-domain spectroscopy [6–9]. The excitation pulses are supplied by a mJ-class Ti:Sapphire regenerative laser (Coherent Libra-HE) generating 76 fs pulses centred at $\lambda = 800$ nm with a 1 kHz repetition rate. The beam diameter (intensity at $1/e^2$) is ≈9mm. The setup comprises two separate beamlines, the THz excitation pump, and the optical sampling probe for detection. By placing a cylindrical neodymium magnet on the side of the spintronic sample, we generate a static magnetic field parallel to the film plane, and therefore, we generate the THz pulse by impinging the femtosecond pulse on the surface of the sample. THz electro-optic detection is implemented by co-propagating the THz field and the optical sampling probe in a standard 1 mm thick <110> ZnTe crystal [10]. The emitted THz wave is measured through a f-f (Fourier) condition on the detection crystal. The detection crystal and the probe polarisation are set to detect the p-polarised THz field. As in standard time-domain spectroscopy schemes, the time-domain traces are reconstructed by varying the delay t_d between the THz pulse and the optical probe. The THz signal is measured with a balanced photodetection unit feeding a lock-in amplifier.

SUPPLEMENTARY DATA AVAILABILITY

Data of the supplementary figures are available from the corresponding author upon reasonable request.

REFERENCES

[1] Björck, M. & Andersson, G. GenX: an extensible X-ray reflectivity refinement program utilizing differential evolution. J Appl Cryst 40, 1174–1178 (2007).

[2] Jin, X. & Han, J. K-Means Clustering. in Encyclopedia of Machine Learning 563–564 (Springer, Boston, MA, 2011). doi:10.1007/978-0-387-30164-8_425.

[3] Miroshnichenko, A. E. et al. Nonradiating anapole modes in dielectric nanoparticles. Nat Commun 6, 8069 (2015).

[4] Malitson, I. H. Interspecimen Comparison of the Refractive Index of Fused Silica*,†. J. Opt. Soc. Am., JOSA 55, 1205–1209 (1965).

[5] Werner, W. S. M., Glantschnig, K. & Ambrosch-Draxl, C. Optical Constants and Inelastic Electron-Scattering Data for 17 Elemental Metals. Journal of Physical and Chemical Reference Data 38, 1013–1092 (2009).

[6] Auston, D. H. & Nuss, M. C. Electrooptical generation and detection of femtosecond electrical transients. IEEE J. Quantum Electron. 24, 184–197 (1988).

[7] Fattinger, Ch. & Grischkowsky, D. Terahertz beams. Appl. Phys. Lett. 54, 490–492 (1989).

[8] Horiuchi, N. Terahertz Technology: Endless applications. Nature Photonics 4, 140–140 (2010).

[9] Seifert, T. et al. Efficient metallic spintronic emitters of ultrabroadband terahertz radiation. Nature Photonics 10, 483–488 (2016).

[10] Wu, Q., Litz, M. & Zhang, X. C. Broadband detection capability of ZnTe electro-optic field detectors. Applied Physics Letters 68, 2924–2924 (1995).

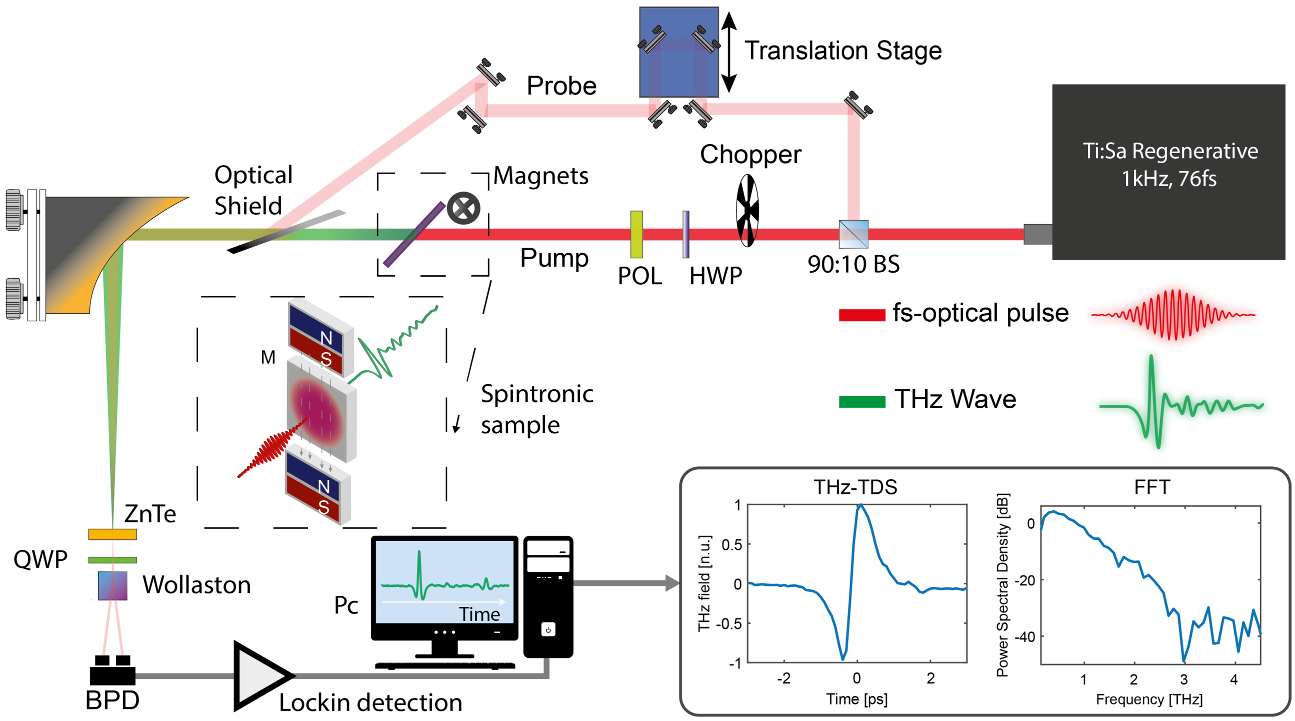

FIG. S7. Scheme of the time-domain spectroscopy apparatus for measuring the ultrafast THz pulses. BS: beam sampler, HWP: half wave-plate, QWP: quarter wave-plate, POL: polariser, ZnTe: zinc telluride, and BPD: balanced photodetectors.
